# Long-Term Follow-Up after Iliosacral Screw Fixation of Unstable Pelvic Ring Fractures

**DOI:** 10.3390/jcm13041070

**Published:** 2024-02-14

**Authors:** Josef Stolberg-Stolberg, Moritz F. Lodde, Dominik Seiß, Jeanette Köppe, René Hartensuer, Michael J. Raschke, Oliver Riesenbeck

**Affiliations:** 1 Department of Trauma-, Hand- and Reconstructive Surgery, University Hospital Muenster, Albert-Schweitzer-Campus 1, Building W 1, 48149 Muenster, Germany; josef.stolberg-stolberg@ukmuenster.de (J.S.-S.); d_seis01@uni-muenster.de (D.S.); michael.raschke@ukmuenster.de (M.J.R.); oliver.riesenbeck@ukmuenster.de (O.R.); 2 Institute of Biostatistics and Clinical Research, University of Muenster, Schmeddingstrasse 56, 48149 Muenster, Germany; jeanette.koeppe@ukmuenster.de; 3 Department of Orthopedics, Trauma-, Handsurgery and Sportsmedicine, Klinikum Aschaffenburg-Alzenau, Am Hasenkopf 1, 63739 Aschaffenburg, Germany; rene.hartensuer@klinikum-ab-alz.de

**Keywords:** pelvic ring injury, iliosacral screw fixation, high-energy trauma, functional outcome, minimally invasive surgery, sacrum

## Abstract

(1) **Background:** High-energy injuries of the pelvic ring are rare. The wide application of iliosacral screw fixation of the posterior pelvic ring is relatively new. The aim of the present study was to evaluate the long-term quality of life. (2) **Methods:** All patients treated with an iliosacral screw for a posterior pelvic ring stabilization after high-energy trauma at a level 1 trauma center between 2005 and 2015 were included. Pelvic ring injuries were classified according to the Tile classification adapted by AO/ASIF. The clinical evaluation included the patient-oriented questionnaires surveys of the Majeed Score, Iowa Pelvic Score (IPS), Work Ability Index (WAI), SF-36, EQ5D-5L. (3) **Results:** A total of 84 patients were included with a median follow-up of 130.1 months (IQR 95.0–162.0 months). The median ISS was 22.5 (IQR 16.0–29.0), mean Majeed Score 83.32 (SD ± 19.26), IPS 77.88 (SD ± 13.96), WAI 32.71 (SD ± 11.31), SF-36 PF 71.25 (SD ± 29.61) and EQ5D-5L 0.83 (SD ± 0.21). There was a notably difference between uni- and bilateral pelvic fractures (*p* = 0.033) as well as a correlation with the ISS (*p* = 0.043) with inferior functional outcome measured by IPS. (4) **Conclusions:** Long-term follow-up of iliosacral screw fixation of unstable pelvic ring fractures showed a good quality of life and functional outcome with equal EQ5D-5L results and inferior SF-36 physical functioning compared to the German population.

## 1. Introduction

High-energy injuries of the pelvic ring are rare with an incidence of 23 to 56 accounting for 0.3–8.2% of all fractures [1]. Due to improved pre-hospital and clinical emergency therapy, such as the use of pelvic binders and intravenous fluid resuscitation, a significantly higher survival rate could be achieved [2]. Early treatment of pelvic ring injuries includes angioembolization, pelvic packing and the pelvic c-clamp [3]. Data of the German Pelvic Trauma Registry showed an in-hospital decrease in mortality [4]. A standard classification system for pelvic ring fractures is the Tile classification adapted by the AO/ASIF (Arbeitsgemeinschaft Osteosynthese/Association of the Study of Internal Fixation) accounting for stability, force direction and patho-anatomy [5,6,7]. In unstable pelvic ring fractures, early surgical fixation is recommended [8]. Letournel’s golden rules states that reduction and restoration of the weight bearing posterior part of the pelvic ring is the primary goal [9]. The use of a percutaneous iliosacral screw is commonly seen as the standard care of surgical fixation [10,11,12]. Before the introduction of this minimally invasive technique, posterior stabilization was performed using posterior and extensive surgical approaches, which are associated with a substantial soft tissue damage [13,14]. Advantages of the iliosacral screw are the minimally invasive surgical technique, the excellent biomechanical characteristics and the safe and effective clinical use [10,15,16,17].

Results of short-term and mid-term follow-up studies after pelvic fractures underline the need for excellent reduction and show a range of functional outcomes with many patients returning to work [18,19,20,21,22]. Chen et al. report in their study on 32 patients who were treated either non-operatively or with iliosacral screws. One year after surgery, they were examined using SF-36 and Majeed score. The non-operative cohort showed poorer results in both questionnaires than the cohort treated with the iliosacral screw. These differences were significant in the sub-dimension SF-36 general health and Work Ability Index (WAI) “Work” and “Sitting”. The pain that the patients were able to indicate using the VAS was also significantly lower after one year in the group treated with iliosacral screws [23]. A total of 30 of the 41 patients in a study by Abhishek et al. were able to return to their previous place of work after at least one year following the trauma. The results in the Majeed score were either “excellent” or “good” in 21 and 13 patients, respectively [24]. Schweitzer et al. reported 66 of 71 patients, who were able to achieve an “excellent” or “good” result after an average follow-up period of 31 months. A total of 86% of patients were able to return to their previous job [25]. Thus, the treatment of pelvic ring fractures using iliosacral screw fixation is a method that enables a good result in terms of function and quality of life in the short term.

Real long-term follow-up >10 years including quality of life of these patients are of immense interest, but not yet available in the literature [26]. The aim of the present study was to evaluate the real long-term outcome after iliosacral screw fixation of unstable pelvic ring injuries. Satisfying results regarding the use of iliosacral screws would confirm the trend of increasing application.

## 2. Materials and Methods

Patient population: All patients treated with an iliosacral screw for a surgical posterior pelvic ring stabilization at a German level 1 trauma center between 2005 and 2015 were included. Minimal follow-up was 75 months. Only adult patients 18 years of age and older were included. The pelvic ring injuries were classified according to the Tile classification adapted by the AO/ASIF analyzing the pre-operative CT scans [5,6]. The fractures were classified independently by two investigators. In case of discrepancy, they were discussed until a consensus was found. Patients with fragility fractures of the pelvis (FFP) due to low energy trauma were excluded.

Baseline data collection: Data of all operated patients were retrieved from the clinical information system using encoded diagnosis for sacral fracture ICD S32.1-89 (International Statistical Classification of Diseases, German Modification; ICD-10 GM) and German procedure classification (OPS) 5.790.0d, 5-798.3 and 5-83.b20. Demographic data included sex, age, ISS, type of treatment and cause of injury. From 367 patients with a pelvic ring injury, 257 met the inclusion criteria. Exclusion criteria were pelvic ring injuries resulting from low energy trauma (fragility fractures of the pelvis, *n* = 27), age < 18 years (*n* = 23), death during treatment period (*n* = 13), incomplete documentation (*n* = 2), external treatment of patient (*n* = 2), operative treatment of pelvic ring fracture other than iliosacral screw (*n* = 32), no pelvic ring fracture (*n* = 3) or other surgical procedure (*n* = 5).

**Figure 1:**
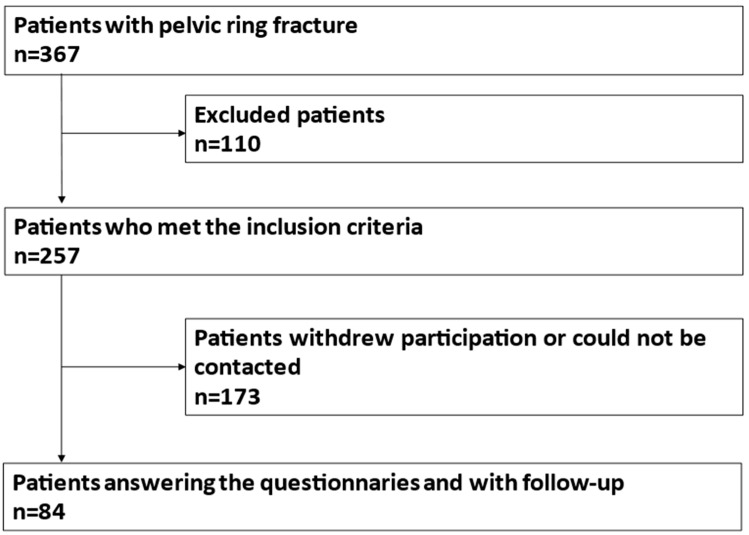
From 367 initially included patients 257 met the inclusion criteria. The questionnaires were answered from 84 patients. The remaining patients refused to participate or could not be contacted.

Assessment of functional outcome and quality of life: Potential participants were contacted by telephone. After agreeing to participate, patients could choose between digital and paper-based participation. In the case of digital participation, patients were sent a letter with a cover letter with a Quick-Response code containing the link to the survey (LimeSurvey, Limesurvey GmbH, Hamburg, Germany), a declaration of consent, a data protection declaration and a pre-addressed and pre-stamped return envelope. If the patients wanted to participate on paper, the questionnaires were sent to them by post in printed form together with the declaration of consent, the data protection declaration and the prepaid return envelope. After at least three unsuccessful contact attempts on different days and times of day, a letter was sent to the last known address with a cover letter, the questionnaires, a pre-addressed and pre-stamped return envelope, a declaration of consent and a data protection declaration. From 257 contacted patients, 84 answered the questionnaires. The remaining patients refused to participate or could not be contacted (Figure 1 PRISMA flow diagram).

The clinical reevaluation included the patient-oriented questionnaires surveys of the Majeed score [27], Iowa Pelvic Score (IPS) [28,29], Work Ability Index (WAI) [30,31,32], Study Short-Form 36-item Health Survey (SF-36) [33] and European Quality of Life 5 Dimensions-5 Level (EQ5D-5L) [34,35]. These scores are established for analyzing functional outcome after pelvic ring injuries and have been used frequently for more than 20 years in similar studies (Table 1) [18,29,30,31,36,37,38,39,40,41,42,43,44,45].

The Majeed score is a pelvic-specific assessment of function after major pelvic injury [27]. It is composed of five factors: pain, standing, sitting, sexual intercourse and work performance. Each factor is determined by one question except for standing, which has three. The possible score ranges from 0 to 100 in order of decreasing disability. The IPS is an instrument to assess functional pelvic specific outcome consisting of six categories worth a total of 100 points: activity of daily living, work history, pain, limp, visual pain line, and cosmesis [28]. The WAI is a tool used in clinical and occupational research to examine work ability [31]. This survey consists of seven questions about the demands of work and the worker’s health status [31]. This score ranges from 7–49. The SF-36 is a general health assessment. This questionnaire survey is based on 36 questions [33]. The EQ5D-5L is a standardized and widely accepted tool to measure health outcome after orthopedic polytrauma injuries [26]. It is composed of five factors: mobility, self-care, pain/discomfort and anxiety/depression as well as a self-rated health scale. A score of 1.0 suggests full health. Reference data from a general population sample are available [26,46].

Statistics: Descriptive data were expressed with means and standard deviation (SD) or median and interquartile range (IQR). Categorical data were exposed as frequencies (%). A confidence interval (CI) of 95% was used. Spearman’s rank correlation coefficient was used for the analysis of the correlations. Multivariable regression models were calculated for continuous target variables using linear regression. Mann-Whitney-U-test was used for comparison of subgroups. The statistical analysis was performed using the SPSS (version 28). A *p*-value of <0.05 was considered as statistically noticeable. All analyses were fully explorative and all results are interpreted accordingly.

Ethics: The study was conducted in accordance with the Declaration of Helsinki and approved by the local ethics committee (Ethics Committee of Westfalen-Lippe, no: 2020-853-f-S).

## 3. Results

Study population and follow-up: The study population (*n* = 84) consisted of 38 female (45.2%) and 46 male (54.8%) patients. Median age at surgery was 47.2 years (IQR 36.3–60.0 years) with a median ISS (Injury Severity Score) of 22.5 (IQR 16.0–29.0). Median age at the time of answering the questionnaire was 58.1 years (IQR 45.0–68.8 years) after a median follow-up of 130.1 months (IQR 95.0–162.0 months).

Trauma mechanism: Car accidents were the most frequent cause for pelvic ring injuries (*n* = 21, 25.0%). Further reasons for pelvic ring injuries were falls from heights >3 m (*n* = 15, 17.9%), motorcycle and cycle accidents (*n* = 8, 9.5% each) and suicidal jumpers (*n* = 3, 3.6%). In 3.6% (*n* = 3) of the cases, pedestrians hit by cars suffered pelvic ring injuries.

Fracture classification: Partially stable AO 61B2 fractures were observed in 23 patients (27.4%) and AO 61B3 fractures in 7 patients (8.3%). Unstable AO 61C1 accounted for 39 patients (46.4%), AO 61C2 fractures for 8 patients (9.5%) and AO 61C3 fractures for 7 patients (8.3%).

Functional outcome and quality of life: The mean Majeed score was 83.32 (SD ± 19.26). The mean IPS of the study population was 77.88 (SD ± 13.96) and the mean WAI was 32.71 (SD ± 11.31). The mean SF-36 PF was 71.25 (SD ± 29.61). The results of the subscales of the SF-36 are shown in Table 2. The comparison of the mean value of this SF-36 PF with the German standard values of the SF-36 PF by Ellert et al. showed a notably difference with inferior outcomes of the presented study population (Wilcoxon test *p* = 0.002) [47]. The age at surgery was negatively correlated with the SF-36 PF (r_s_ = −0.497; 95% CI [−0.929, −0.066]; *p* = 0.024) as well as strong negatively correlated with the SF-36 RP (r_s_ = −0.933; 95% CI [−1.564, −0.302]; *p* = 0.004, multivariable regression). The other tested parameters: number of months after surgery, sex, age at surgery in years, ISS divided into <16 and ≥16, pelvic ring fracture B or C and pelvic ring fracture unilateral or bilateral had no significant influence on SF-36 PF and SF-36 RP.

The average EQ5D-5L index was 0.83 (SD 0.21). The following influencing parameters were included in a multivariable linear regression of the dependent variable EQ5D-5L index: The number of months after surgery, sex, age at surgery in years, ISS divided into <16 and ≥16, pelvic ring fracture B or C and pelvic ring fracture unilateral or bilateral. None of the influencing variables mentioned had a notably effect on the EQ5D-5L index in this model. The comparison of the mean value of between the analyzed cohort and the German standard values of the EQ5D-5L index by Grochtdreis et al. showed no difference (Wilcoxon test *p* = 0.910) [48].

No difference was observed between type of fracture and unilateral or bilateral fracture respectively on quality of life (*p* > 0.05 Mann-Whitney U Test). However, bilateral pelvic fractures showed a noticeably lower IPS (*p* = 0.033) (Table 3).

EQ5D-5L (Mean 0.82 vs. 0.83, *p* = 0.476) and SF 36 Physical Function (Mean 69.67 vs. 72.13, *p* = 0.600) between the partial stable pelvic ring fractures (AO 61B) and the unstable pelvic ring fractures (AO 61C) were not statistically different.

## 4. Discussion

The aim of the present study was to evaluate the long-term quality of life after iliosacral screw fixation of unstable, high-energy pelvic ring fractures. The most important results show that the functional long-term outcome and quality of life after iliosacral screw fixation in high-energy pelvic ring injuries is good to excellent. The data underline the effectiveness of the surgical technique for stabilization of pelvic ring injuries.

It is still not fully understood, which factors affect the functional outcome after high-energy pelvic ring injuries [37]. Dysfunction of the pelvic ring after trauma can lead to functional limitations of non-specific daily activities like walking, standing, sitting and disbalance of the lumbosacral region [49]. Furthermore, neurological dysfunction can cause severe morbidity and has a significant negative prognostic value of quality of life [50,51,52]. Hence, early anatomic reduction and stabilization may be associated with an improved neurological recovery [50]. However, there is still no functional outcome score which exclusively values the impairment of function of the pelvis after a pelvic ring injury [37,53]. Generic instruments are useful for several medical conditions [54,55]. Specific aspects of the injury are not addressed and studies examining the functional outcome and quality of life after pelvic ring injuries use several different scores with poor level of evidence [4,22,40,42,53,56,57].

A systematic review of the literature analyzing outcome measurements after surgical treatment of pelvic ring injuries observed that the most commonly used generic score is the SF-36 [53]. The most common used pelvic-specific scores were the Majeed score and the IPS [53]. The SF-36 is a generic score and effective describing the patient`s level of emotional status, pain and general disability with an age- and gender-matched set of normal values available [29,36,40]. Our data showed a reduced SF-36 physical functioning (71.25 points) compared to the German population (86.6 points) [47]. This is consistent with previously published data [58]. Many patients with unstable pelvic ring injures suffer from concomitant injuries [4,56,57]. The functional outcome may be also affected by these injuries, being a possible bias of the analysis of functional outcome [40,56]. For that reason, we performed a multivariable regression and Spearman’s rank correlation. In our study, we could show that the type of injury and the ISS were associated with a worse functionality measured by IPS but not long-term quality of life, which is also consistent with several earlier published studies and important for patient consultation [18,37,43,56,59].

It is emphasized that anatomic reduction and internal fixation of pelvic ring injuries is mandatory for a good outcome. Displacement of the posterior pelvic ring more than 1 cm is associated with a worse long-term outcome [60,61]. Internal fixation of unstable pelvic ring fractures leads to good functional outcome [10,22,56]. Especially the anatomic exact reduction in sacroiliac dislocation is important for a high functional success rate [18,43]. Contrary, another previously published study showed that the degree of vertical displacement after surgical fixation did not have an effect on functional outcome [29]. Rommens and Hessmann reported that functional outcome is inferior for patients with type C fractures of the pelvic ring and Pohlemann et al. showed that only 27% of patients with type C pelvic ring injures have a good or excellent functional outcome [57,59]. However, Miranda et al. examined no differences in functional outcome regardless of the fracture type [39], which is in contrast with the results of the present study. The Majeed score and IPS of the present study cohort after a 10 years was very similar to the study results of Suzuki et al. [37], who examined the functional outcome after unstable pelvic ring fractures with a minimum follow-up of only two years. In the present study, a correlation between the ISS and the functional outcome could be observed in the IPS score. Patients with bilateral fractures showed significantly inferior results in the IPS. These findings are in accordance with previous published results [37]. In our opinion this underlines the importance of the anatomic reposition, stable fixation and accurate surgical technique for achieving sufficient functional outcome after pelvic ring injuries [10,60,61].

The limitations of this study include the retrospective nature of single-center data collection and the lack of an age-matched control population. Furthermore, only 84 out of 257 participants were included. Patients who were dissatisfied with their outcome might have been unwilling to participate in the study, which might be a cause of bias. Patients who died later in the course or prehospital were also unable to take part, which might have had the same effect. Additionally, a traumatic pelvic ring fracture is often only one of many other injuries suffered by a polytraumatized patient. As a result, this can also have an influence on the feasibility of the tasks required and therefore also on the results of the study. Future studies could employ with finite element simulation analysis to explore the effects of factors such as anatomic differences between populations, postoperative conditions, and postoperative mobilization on the internal biomechanics [62]. It has been shown that the Majeed score and IPS correlate with the SF-36 [53]. Yet, the overall validity, reliability and responsiveness of these widely used instruments for measuring functional outcome after pelvic injuries have not been established [53]. However, the time period of 10 years provides data of real long-term functional follow-up after unstable pelvic ring factures treated with iliosacral screw insertion. To our best knowledge this was not shown before.

## 5. Conclusions

Unstable pelvic ring fractures are severe injuries with a significant impact on functional status and quality of life. The quality of life of patients with iliosacral screw fixation after traumatic pelvic ring fracture is partially comparable to that of the normal population. In the SF-36, the participants in this study were significantly inferior to the normal population. Age at the time of trauma had a noticeable influence in multivariable regression analyses of the SF-36, especially in physical scales such as the SF-36 PF, and could therefore represent a risk factor for a poorer long-term outcome. The EQ5D-5L showed no difference in quality of life compared to the German norm data. According to the WAI conducted in this study, the patients’ ability to work was rated as moderate in patients under 65 years of age. Nevertheless, 67.6% of the patients were able to return to their old workplace with the same or reduced workload. The present study demonstrates that after 10 years of follow-up, a moderate to good functional outcome and quality of life can be achieved after iliosacral screw fixation of high energy pelvic ring injuries. Future studies need to analyze clinical data and functionality in a prospective manner with an age matched cohort.

## Figures and Tables

**Table 1:** Overview of assessed scores and their content including EQ5D-5L, SF-36, MPS, IPS and WAI.

EQ5D-5L	SF-36	MPS	IPS	WAI
Standardized and widely accepted tool to measure health outcome after orthopedic polytrauma injuries. Based on 5 dimensions each with 5 levels.	General health assessment. Based on 36 questions and divided into 8 subscales.	Pelvic-specific assessment of function after major pelvic injury including five categories: Sitting, pain, work, sexual intercourse, mobility.	Instrument to assess functional pelvic specific outcome consisting of six categories: activity of daily living, work history, pain, limp, visual pain line, and cosmesis.	Tool of clinical and occupational research to examine work ability including general health questions and subjective assessment of the ability to work.

**Table 2:** The subscales of SF-36.

SF-36 General Health (GH)	Mean 63.06 (SD 22.87)
SF-36 Physical Functioning (PF)	Mean 71.25 (SD 29.61)
SF-36 Bodily pain (BP)	Mean 71.49 (SD 29.50)
SF-36 Vitality (VT)	Mean 57.44 (SD 22.38)
SF-36 Mental Health (MH)	Mean 72.34 (SD 20.73)
SF-36 Social functioning (SF)	Mean 81.48 (SD 23.78)
SF-36 Role-functioning physical (RP)	Mean 66.98 (SD 44.34)
SF-36 Role-functioning emotional (RE)	Mean 80.25 (SD 34.47)

**Table 3:** There were no differences in quality of life in the 10 years follow-up outcome between partial stable pelvic ring fractures (AO 61B) and unstable pelvic ring fractures (AO 61C). Bilateral pelvic fractures showed a notably lower IPS. The IPS was also correlated with the ISS.

Score	Mean of Subgroup Fracture Classification		*p* Value (Man-Whitney-U-Test)	Mean of Subgroup Uni- vs. Bilateral Fractures		*p* Value (Man-Whitney-U-Test) * Indicates a Significant Value (p < 0.05)	Correlation (Spearman’s-Rank Correlation)
	AO 61B	AO 61C		Unilateral	Bilateral		ISS Correlation Coefficient (*p*-Value)
EQ5D-5L	0.82	0.83	0.476	0.85	0.79	0.431	−0.061 (0.584)
SF 36 Physical functioning	69.67	72.13	0.600	72.42	67.95	0.662	−0.105 (0.340)
SF 36 Role emotional	81.61	79.49	0.741	80.00	80.95	0.644	0.072 (0.520)
SF 36 Role physical	69.64	65.57	0.878	70.00	58.33	0.258	0.013 (0.908)
SF 36 Vitality	55.50	58.52	0.512	56.77	59.32	0.725	0.061 (0.579)
SF 36 Mental Health	73.20	71.85	0.703	72.13	72.91	0.756	−0.014 (0.903)
SF 36 Social functioning	77.92	83.49	0.119	82.17	79.55	0.771	−0.037 (0.737)
SF 36 Bodily pain	74.58	69.77	0.467	73.43	66.02	0.354	−0.127 (0.251)
SF 36 General Health	62.93	63.14	0.960	63.10	62.95	0.957	−0.116 (0.304)
WAI	31.97	33.08	0.399	32.79	32.45	0.874	−0.116 (0.448)
MPS	86.52	81.69	0.533	84.88	78.25	0.193	−0.230 (0.060)
IPS	78.88	77.32	0.822	79.56	73.11	0.033	−0.237 * (0.043)

## Data Availability

Data will be sent on request.
